# A Reliable Health Indicator for Fault Prognosis of Bearings

**DOI:** 10.3390/s18113740

**Published:** 2018-11-02

**Authors:** Bach Phi Duong, Sheraz Ali Khan, Dongkoo Shon, Kichang Im, Jeongho Park, Dong-Sun Lim, Byungtae Jang, Jong-Myon Kim

**Affiliations:** 1 School of Computer Engineering and Information Technology, University of Ulsan, Ulsan 44610, Korea; duongbachphi@gmail.com (B.P.D.); sherazalik@gmail.com (S.A.K.); dongkoo88@gmail.com (D.S.); 2 ICT Convergence Safety Research Center, University of Ulsan, Ulsan 44610, Korea; kichang@ulsan.ac.kr; 3 Industry IT Convergence Research Group, Intelligent Robotics Research Division, SW Contents Research Laboratory, Electronics and Telecommunications Research Institute (ETRI), Daejeon 34129, Korea; parkjh@etri.re.kr (J.P.); dslim@etri.re.kr (D.-S.L.); jbt@etri.re.kr (B.J.)

**Keywords:** gradient analysis, health indicator, prognosis and health management, remaining useful life, rolling element bearing, wavelet packet transform

## Abstract

Estimation of the remaining useful life (RUL) of bearings is important to avoid abrupt shutdowns in rotary machines. An important task in RUL estimation is the construction of a suitable health indicator (HI) to infer the bearing condition. Conventional health indicators rely on features of the vibration acceleration signal and are predominantly calculated without considering its non-stationary nature. This often results in an HI with a trend that is difficult to model, as well as random fluctuations and poor correlation with bearing degradation. Therefore, this paper presents a method for constructing a bearing’s HI by considering the non-stationarity of the vibration acceleration signals. The proposed method employs the discrete wavelet packet transform (DWPT) to decompose the raw signal into different sub-bands. The HI is extracted from each sub-band signal, smoothened using locally weighted regression, and evaluated using a gradient-based method. The HIs showing the best trends among all the sub-bands are iteratively accumulated to construct an HI with the best trend over the entire life of the bearing. The proposed method is tested on two benchmark bearing datasets. The results show that the proposed method yields an HI that correlates well with bearing degradation and is relatively easy to model.

## 1. Introduction

Prognosis and health management of rotary machines is an important research area. Generally, this involves condition monitoring using appropriate sensors, assessment of the current health status of the machines and predicting their future health by analyzing acquired measurement data, and utilization of this knowledge to improve the overall reliability and availability of the machines [1,2,3,4,5,6,7,8,9]. Rolling element bearings are among the most significant contributors to the failure of critical industrial equipment, such as induction motors [10,11]. Consequently, they have received considerable research attention [2,3,4,5,7,8,12,13]. Most of the research conducted on bearing health prognosis has involved the development of techniques to estimate the remaining useful life (RUL) of bearings. This cannot be accomplished with absolute precision due to the inherent acausal nature of the problem [1,14,15]. That is, the bearing’s RUL depends on future operating conditions and load profiles, which cannot be precisely determined in advance; thus, they are assumed to be stationary. Nevertheless, a reasonable estimate of a bearing’s RUL is helpful for scheduling maintenance and avoiding abrupt machine failures.

There are four important aspects related to the problem of bearing health prognosis, as identified in an extensive and systematic review [7] of the most recent literature on the topic. Three of these aspects are the availability of appropriate and representative measurement data, the construction of an appropriate health indicator (HI) that can be used to infer the true health of the bearing, and the detection of different health stages through which a bearing evolves during its lifetime using the HI. This last aspect involves determining the time-to-start prediction (TSP) or the first prediction point (FPT), which is the point at which a bearing departs from its normal healthy behavior and begins showing signs of degradation. The failure threshold for the HI indicates the bearing’s end of life (EOL). The last important aspect is RUL estimation of the bearing, which is done by developing models for the evolving trends in the bearing HI and using those models to project the HI until the bearing reaches its EOL. These aspects are briefly discussed below. The primary contribution of this study is the construction of a bearing HI; this is discussed in detail in Section 2.

Since it is difficult to acquire run-to-failure test data for bearings in an industrial environment, most studies on bearing health prognosis [2,3,4,5,8,9,12,13] have been conducted using accelerated degradation or run-to-failure test data collected under laboratory conditions. These data are typically collected using accelerometers that measure the vibration acceleration of bearing housings [9,16,17,18,19]. Additionally, some researchers have used acoustic emission data (AE) to estimate the RUL of bearings [4]. Two such datasets that are freely available to the research community and have been extensively used for research on different aspects of bearing health prognosis are the Intelligent Maintenance Systems (IMS) dataset [16,17] and the PRONOSTIA dataset [18,19]. The useful lives of the bearings employed in the collection of the IMS data ranged from 7 to 35 days, whereas the bearings in the PRONOSTIA platform had useful lives of only several hours. These datasets have notable differences and pose different challenges to researchers, as discussed in [7].

Essentially, bearing health prognosis is concerned with the quantitative assessment and prediction of damage to a bearing over its operational life. Since it is prohibitively difficult to directly observe and quantify the damage that a bearing or any of its components undergoes during operation, this assessment must be made through measurement data, i.e., the vibration acceleration [2,3,5,8,9,12,13] or the AE signals [4]. Generally, one of the features of the measurement data is used as an HI to infer the true health of the bearing. The selection or construction of an appropriate HI is very important to correctly determine both the onset and progression of degradation in bearings. A good HI should correlate well with the physical degradation of a bearing, which is assumed to be irreversible [7]. Thus, an HI is expected to exhibit a monotonically increasing or decreasing trend, which is determined by various metrics, such as the monotonicity [5,20,21,22]. Moreover, the HI should be robust to noise and stochastic fluctuations, which is not always the case. Hence, researchers have used different smoothing techniques to remove noise and spurious fluctuations in the HI [2,4,5,22,23] or improve the signal-to-noise ratio [24,25].

The root mean square (RMS) value of the time-domain vibration acceleration signal is a commonly used HI [2,8,23,26]. Nevertheless, researchers have experimented with other choices for HIs, and different techniques have been employed to construct HIs that correlate well with a bearing’s degradation, i.e., an HI shows a monotonically increasing or decreasing trend and is robust to noise and random fluctuations. For instance, Singleton et al. used the variance of the time-domain vibration signal [13] as an HI. Elforjani et al. [4] proposed using the signal intensity estimator as the bearing HI, which is a scaled ratio of the sum of cumulated sums of a segment of an AE signal to the entire length of the AE signal. Javed et al. [5] proposed using trigonometric functions and cumulative or pointwise running sums of features with appropriate scaling to construct an HI with better monotonicity. Deutsch et al. [9] used the values of the fast Fourier transform (FFT) of the vibration signals to estimate the RUL of a bearing. Liao et al. [22] used a genetic algorithm to construct an HI using different combinations of features and different mathematical formulations. Jin et al. [3] assessed the health of a bearing using Mahalanobis distances from wavelet features of bearings in healthy states. Guo et al. [27] proposed a recurrent neural network (RNN)-based HI, which is constructed by selecting sensitive features from an initial set of 14 features using metrics such as the monotonicity and correlation; those features are then fused using the RNN to yield an RNN-HI. Malhi et al. [28] used the RMS and peak values of the wavelet coefficients as the HI for bearings. In [29], the authors used the kurtosis of band pass-filtered vibration signals as the HI. Similarly, Li et al. [30] used the mathematical morphology pattern spectra of vibration signals as the HI, whereas Loutas et al. [31] used the spectral flatness as the HI for bearings. The reviews in [7,32] provide a more comprehensive overview of the health indicators used for the health prognosis of rotary machines in general and bearings in particular.

Despite the above developments, finding an HI that is physically practical, reveals the bearing degradation, and is grounded in well-established principles remains challenging. The vibration acceleration and AE signals that are recorded from bearing housings are inherently nonstationary, and the transient activity caused by developing faults can be effectively captured by analyzing these signals at different resolutions in the time-frequency domain [33,34]. This has prompted researchers to use time-frequency analysis techniques, such as sparsogram [35] and the discrete wavelet packet transform (DWPT), for bearing fault diagnosis [34,36,37]. In this paper, we propose a method of analyzing the vibration acceleration signals at the sub-band level. Instead of directly calculating the HI value for the raw signal, we first decompose the raw vibration acceleration signal into different sub-bands. The HI is then calculated from each of these sub-band signals. The RMS value is used as the HI; however, it is not directly calculated from the raw signal. The trend in the HI over the lifetime of the bearing varies across different sub-bands. This aspect is illustrated in Section 4. For some sub-bands, the HI shows a monotonically increasing trend, whereas it shows fluctuations and increasing–decreasing–increasing trends for others. At each time index, we evaluate the HIs extracted from all of the sub-bands by using a gradient-based method. The sub-band with the maximum gradient is selected to construct the optimal HI, which exhibits a monotonically increasing degradation trend and is robust to noise and random fluctuations. In short, the main contributions of this study are:

1) A novel method is proposed to construct a bearing HI through sub-band analysis of the vibration acceleration signals, which are inherently nonstationary and require analysis at different resolutions in the time-frequency domain to capture the maximum amount of information related to bearing degradation. An HI that is calculated for the entire raw signal fails to capture this information. Alternatively, decomposing the signal into different sub-bands enables analysis and selection of the best available information that is related to bearing degradation from individual sub-bands.
2) A gradient-based metric is proposed to evaluate the HI extracted from individual sub-bands at each time index. The sub-bands that exhibit the best trend in terms of the proposed metric are then selected to construct an optimal HI that can be used to infer a bearing’s health and estimate its RUL.
3) The proposed method yields an HI that monotonically increases and is robust to random fluctuations and noise. The RMS value is used as the HI; it is calculated using a novel method and can be used to infer the true level of physical degradation in the bearing and estimate its RUL.

The remainder of this paper is organized as follows: in Section 2, the datasets used in this study are briefly introduced to validate the proposed method. In Section 3, the proposed method for the construction of the HI is detailed and illustrated. In Section 4, the results are presented and discussed, and our conclusions are presented in Section 5.

## 2. Accelerated Bearing Degradation Test Data

The proposed method for the construction of an HI for bearing health prognosis was tested on two of the most widely used accelerated bearing degradation test datasets that are available in the National Aeronautics and Space Administration Ames Research Center data repository, i.e., the IMS dataset, which was published by Intelligent Maintenance Systems at the University of Cincinnati [16,17], and the PRONOSTIA dataset, which was published by Franche-Comté Electronics Mechanics Thermal Science and Optics-Sciences and Technologies (FEMTO) [18,19]. Details of the experimental testbeds that were used to acquire these datasets are provided in the relevant works listed in the reference section, while the salient features of these datasets are described below.

The IMS dataset contains data for three accelerated degradation tests. For each test, four Rexnord ZA-2115 double-row bearings were installed on a single shaft with no seeded defects. The durations of the tests ranged from 7 to 35 days. Vibrations of the bearings were measured using accelerometers installed along the horizontal and vertical axes. The vibration acceleration was measured every 10 min for 1 s at a sampling rate of 20 kHz. Thus, after each 10-min measurement interval, 20,480 data points of the vibration acceleration were recorded. Most of the bearings in this dataset showed an increasing–decreasing–increasing degradation trend, suggesting the development and subsequent healing of an incipient defect before the onset of severe damage in a bearing [7,17].

The PRONOSTIA dataset contains data for 17 run-to-failure tests that were performed under three different operating conditions, each using one roller element bearing. All of these degradation tests lasted for shorter durations than those of the IMS dataset, i.e., only a few hours. The vibration acceleration was measured using two accelerometers installed along the horizontal and vertical axes of the bearing. The vibration signals were recorded every 10 s for a duration of 0.1 s at a sampling rate of 25.6 kHz, resulting in a very low frequency resolution for this dataset. Thus, after each 10 s measurement interval, 2560 data points of the vibration acceleration were recorded. The PRONOSTIA dataset is considered very challenging for bearing health prognosis because most of the bearings experience sudden accelerated degradation, unlike the IMS dataset, and the bearing degradation behavior widely varies, even for the same operating conditions [7]. The challenges posed by both of these datasets make it that much more important to find an HI that can reveal true bearing degradation under different operating conditions.

## 3. Proposed Methodology for the Construction of a Bearing Health Indicator

**Figure 1:**
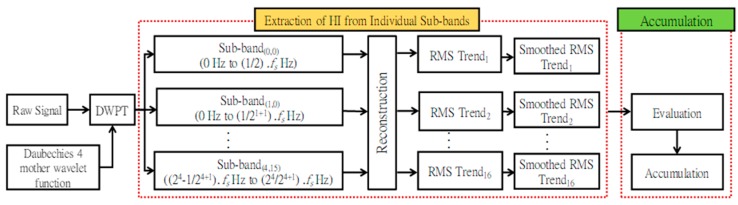
Proposed methodology for the construction of a bearing HI using sub-band analysis of the vibration acceleration signal.

The proposed method for constructing a bearing HI is illustrated in Figure 1. It consists of two distinct processes. The first process involves decomposition of the raw vibration acceleration signal into different sub-bands using the DWPT, followed by the extraction and smoothing of the RMS (HI) values from individual sub-bands. The second process involves evaluation of the RMS trends extracted from individual sub-bands at each time index using the proposed gradient-based method.

Once the RMS trend quality is determined for each sub-band at each time index, the best RMS trends are selected from different sub-bands and accumulated for the entire duration of the bearing operation. The result is an optimal HI that shows the best trend, i.e., a monotonically increasing trend, at all times during bearing operation. These processes are detailed in the subsection below.

### 3.1. Extraction of a Health Indicator from Individual Sub-Bands

The vibration acceleration signal is inherently non-stationary [33,34,36], and the RMS value of the raw signal may not always reveal the true degradation trend because it can miss important fault information that is spread across different time-frequency locales. In short, the RMS value calculated for the raw vibration signal aggregates information from both good and bad sub-bands of the signal into one number, thereby compromising the overall quality of the information extracted from the signal.

The above process results in RMS trends that do not always show a monotonically increasing trend; consequently, they may not correlate well with bearing degradation. The process may also result in RMS trends that are less robust to noise and exhibit large random variations. Hence, in this paper, we decompose the raw vibration acceleration signal into different sub-band signals using the DWPT and then extract the RMS from each sub-band signal. These extracted RMS trends from different sub-bands are then evaluated to select the best trends for the construction of the final health indicator.

The DWPT [38] is an extension of wavelet decomposition and a generalization of the discrete wavelet transform. It is a powerful mechanism for signal processing and has been effectively used for bearing fault diagnosis [34,36]. Compared to normal wavelet analysis, the DWPT can analyze a given signal at better frequency resolutions [39], making it more useful for analyzing non-stationary signals than other techniques that are commonly used for bearing fault diagnosis and prognosis [40,41,42].

As shown in Figure 1, the raw vibration signal is first decomposed into 16 sub-bands using a four-level DWPT and the Daubechies-4 mother wavelet function [34,36]. The parameters of the DWPT, i.e., the number of sub-bands or the level of decomposition of the vibration acceleration signal, and the mother wavelet function are similar to the ones proposed in [34,36]. These sub-bands are then reconstructed based on the coefficients of the frequency bands, and the RMS values are calculated for each reconstructed sub-band signal. This results in 16 RMS values for a single time index. The process is repeated for each time index, and as a result, 16 different RMS curves or trends are obtained, one corresponding to each sub-band signal.

### 3.2. Smoothing of the Health Indicator

The RMS curves obtained from different sub-band signals may exhibit random fluctuations. Researchers have utilized different smoothing and curve-fitting techniques to remove small, random fluctuations in the HI curve and reveal the true degradation behavior [2,4,5]. Bearing degradation is assumed to be an irreversible process that always increases with time; hence, the HI is expected to always show an overall increasing trend with time. This seems like a reasonable assumption, and it makes modeling the trends of the HI easier. However, in the IMS dataset, an increasing–decreasing–increasing trend is observed in the HIs of most bearings. Some researchers have attempted to explain this by alluding to the phenomenon of self-healing in bearings [7,17]. Common smoothing techniques do not filter such trends from the HI; rather, these techniques are only used to reduce the small, local fluctuations in the HI. In this work, the RMS curves obtained from the 16 sub-bands are smoothened using locally weighted regression (LOESS) [43]. In LOESS, every smoothed value is determined using the neighboring data points within a given span. The regression weight for each data point in the span is calculated using Equation (1):

(1) $$w_{i}={\left(1-{\left|\frac{x-x_{i}}{d(x)}\right|}^{3}\right)}^{3}$$

Here, *x* is the predictor value associated with the value to be smoothened, *x_i_* denotes the nearest neighbors of *x* in the span, and *d*(*x*) represents the horizontal distance between *x* and the most distant predictor value in the span. LOESS then uses locally quadratic regression as a weighted linear least-squares regression, determining the smoothed value through weighted regression at the predictor value.

### 3.3. Evaluation and Accumulation of Health Indicators from Individual Sub-Bands

**Figure 2:**
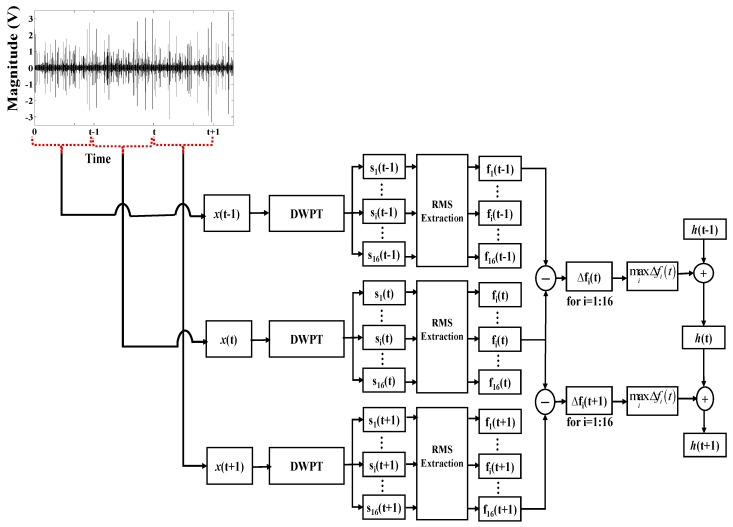
Process of evaluating the RMS trends obtained from different sub-bands and the construction of the optimal health indicator.

Once the HI curves are extracted from individual sub-bands and smoothened, these curves are evaluated at each time index to determine the sub-band that exhibits the best trend. These trends are then accumulated over the entire operational life of the bearing. This process is illustrated in Figure 2, which shows three vibration acceleration signals that are recorded at three different time indices. The three signals are each decomposed into 16 sub-band signals using the DWPT. For each sub-band signal, and at each time index, the RMS value is calculated and smoothened. Thus, at each time index, we have 16 smoothened RMS values, denoted as $f_{1}\left(t\right),f_{2}\left(t\right),\ldots ,f_{16}\left(t\right)$, with one corresponding to each sub-band. Then, for each sub-band, the gradient of the RMS values is determined by calculating the difference between the RMS values for successive time indices, as given in Equation (2):

(2) $$\underset{i=1,2,\ldots 16}{\Delta f_{i}}=f_{i}\left(t+1\right)-f_{i}\left(t\right)$$

Then, at each time index, the maximum value among the gradients for the 16 sub-band signals is determined. This maximum gradient value represents the best trend of the RMS values among all of the sub-bands at a particular time index. It is used to construct the optimal value of the HI using Equation (3):

(3) $$h\left(t+1\right)=h\left(t\right)+\underset{i=1,2,\ldots ,16}{\max\;\Delta f_{i}}$$

Here, *h*(*t*) is the value of the health indicator at time *t* and $\Delta f_{i}$ is the gradient of the *i*th sub-band at time *t*. This process is repeated at each time index to generate the HI curve with the best trend for the entire duration of bearing operation.

## 4. Results and Discussion

In this section, the results of the proposed method for the construction of a bearing health indicator are presented and discussed for the IMS and PRONOSTIA run-to-failure test datasets, respectively.

### 4.1. HI Trends for the IMS Dataset

**Figure 3:**
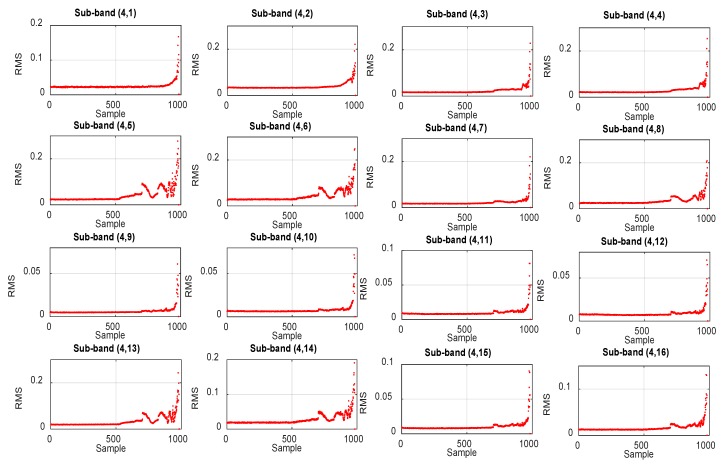
RMS trends extracted from 16 sub-bands of the vibration acceleration signals for bearing 1 of the second run-to-failure test of the IMS dataset, which fails owing to an outer race failure.

**Figure 4:**
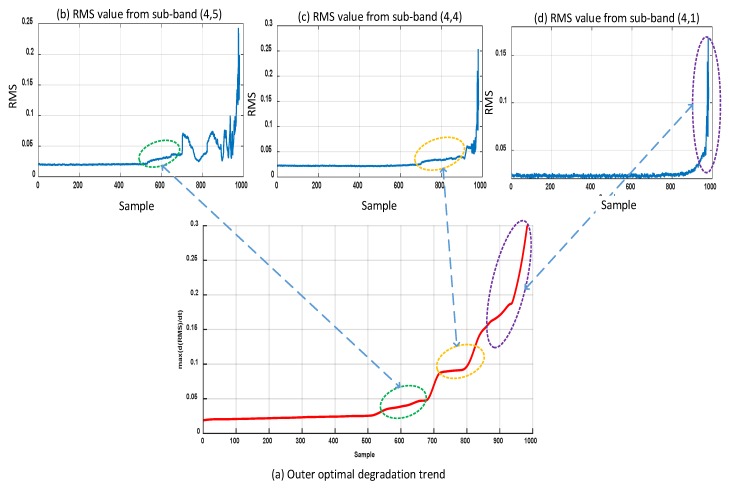
Process of accumulating the best trends in the RMS values from different sub-bands for bearing 1 of the second run-to-failure test of the IMS dataset, which failed owing to an outer race failure: (**a**) Outer optimal degradation trend, (**b**) RMS value from sub-bands (4,5), (**c**) RMS value from sub-band (4,4), (**d**) RMS value from sub-bands (4,1).

Figure 3 demonstrates the effectiveness of using sub-band analysis for the extraction of the HI. The RMS trends in Figure 3 correspond to 16 sub-bands of the vibration acceleration signals for bearing 1 of the second run-to-failure test of the IMS dataset. This particular bearing failed due to failure of its outer race. It is observed in Figure 3 that the behavior of the RMS values is different in different sub-bands. For some sub-bands, it shows a monotonically increasing trend throughout the bearing lifetime; alternatively, for others, it shows fluctuations. Moreover, some sub-bands are more useful in detecting incipient degradation, whereas others show no signs of incipient degradation. The basic idea of the proposed method is to combine the best of each sub-band at each time index and construct an HI that accurately captures bearing degradation throughout its lifetime. This process of accumulating the best trends from different sub-bands of the vibration acceleration signal is detailed in Section 3. The same process is illustrated in Figure 4, which shows how good trends from different sub-bands of the vibration acceleration signal are accumulated to construct the optimal HI with a monotonically increasing trend. Figure 3 and Figure 4 show results for the same bearing.

**Figure 5:**
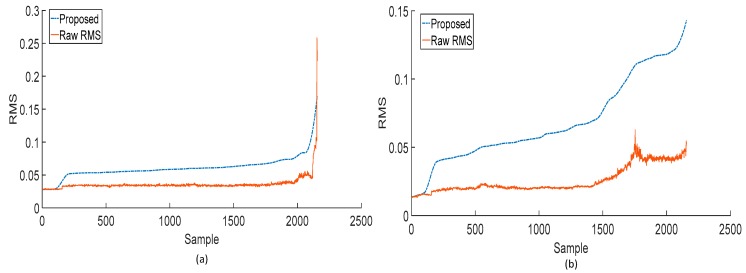
Comparison of RMS trends extracted directly from the raw vibration signals and RMS trends extracted using the proposed method for (**a**) bearing 3 and (**b**) bearing 4 of the first run-to-failure test of the IMS dataset.

Similarly, Figure 5 shows a comparison of the RMS values extracted directly from the raw vibration acceleration signals and the RMS values extracted using the proposed method. Both the comparison of RMS trends in Figure 5 and the RMS trend shown in Figure 4 show that the RMS trends extracted using the proposed methods present a monotonically increasing degradation trend. As mentioned earlier and apparent in Figure 4 and Figure 5, the RMS values extracted from the raw vibration acceleration signals of the IMS dataset show an increasing–decreasing–increasing behavior. Through analysis of the vibration signals at the sub-band level, the proposed method reveals the true degradation in the bearing.

### 4.2. Effect of Smoothing on the RMS Trends

**Figure 6:**
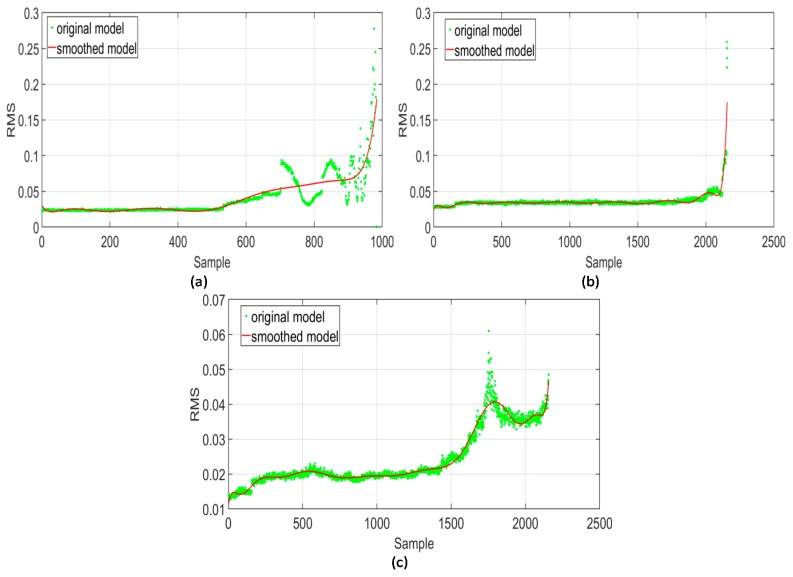
Effect of smoothing using locally weighted regression on the RMS trends of (**a**) bearing 1 of the second run-to-failure test, (**b**) bearing 3 of the first run-to-failure test, and (**c**) bearing 4 of the first run-to-failure test. The IMS dataset was used for these tests.

A bearing’s health indicator, whether it is extracted from the raw signal directly or from any of its sub-bands, almost always exhibits an underlying long-term trend and high-frequency spurious fluctuations that are mostly local in nature. To estimate a bearing’s RUL, revealing the true long-term trend of the health indicator is highly desirable. Accurate estimation of a bearing’s RUL requires the removal of high-frequency, spurious fluctuations in the health indicator. The primary purpose of smoothing is to filter out these fluctuations from the health indicator curve to reveal its true long-term trend. Figure 6 illustrates the effect of smoothing on the RMS curves extracted from different sub-bands of three bearings from the IMS dataset. Smoothing via locally weighted regression removes spurious local fluctuations from the RMS curve and highlights its long-term trend. The LOESS method is applied with a span value of 0.1, resulting in a curve that best fits the RMS values extracted from each sub-band signal. Smoothing helps remove any sharp changes in the gradient of the RMS values that may have been caused by noise or stochastic variations in the degradation process. This enables the proposed scheme to generate HI trends that are easier to model and yield better RUL estimates, while also reducing the effects of noise and random variations.

### 4.3. HI Trends for the PRONOSTIA Dataset

The PRONOSTIA dataset is considered to be a very challenging dataset for estimating the RUL of bearings [2,7] because many algorithms do not achieve good results with these data [2,8,13]. This difficulty stems primarily from the inability of RUL estimation algorithms to correctly model the degradation behavior of bearings. HIs extracted directly from the raw vibration signals—the RMS values [2,8], variance, or Renyi entropy [13]—exhibit highly nonlinear behavior that is inconsistent across bearings, even under the same operating conditions. Moreover, the transition of the bearing from a normal, healthy state to a state of severe degradation is fairly sudden in most of the cases, and many times the trend in the HI does not correlate well with the expected degradation in the bearing. One way to improve this situation is to construct a health indicator that is more indicative of the degradation that a bearing actually experiences. In the proposed method, we strive to achieve this objective by using sub-band analysis of the vibration acceleration signals, as discussed in the preceding sections. The resulting HI exhibits a monotonically increasing behavior throughout the bearing’s lifetime. It is also free of random fluctuations and misleading trends.

**Figure 7:**
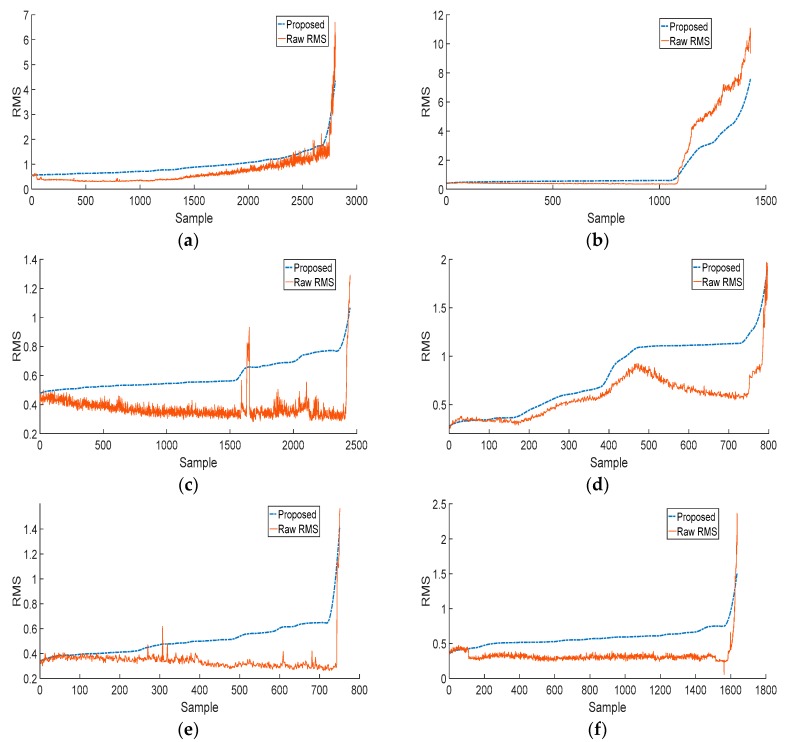
Comparison between the trend in RMS extracted directly from the vibration acceleration signal and the one extracted using the proposed method for (**a**) bearing 11, (**b**) bearing 14, (**c**) bearing 16, (**d**) bearing 22, (**e**) bearing 24, and (**f**) bearing 32 of the PRONOSTIA dataset.

Figure 7 shows the RMS trends for six different bearings of the PRONOSTIA dataset that were extracted using the proposed method, comparing them with the RMS values calculated directly for the raw vibration signals, which is a common procedure. Most of these bearings, except for bearing 11 in Figure 7a, exhibit RMS trends that are very difficult to model and hence result in poor RUL estimates. The RMS values extracted using the proposed method yield better HI trends that are easier to model and yield better RUL estimates. Thus, the proposed method yields an HI that clearly reveals the bearing degradation and exhibits a trend that is easier to model for RUL estimation.

### 4.4. Quantitative Evaluation of the Proposed Health Indicator

The performance of the proposed method for calculating the bearing health indicator is compared with methods based on the variance [13] or conventional RMS, which are popular choices for bearing health indicators; conventional RMS is calculated directly from the raw vibration acceleration signal without any sub-band analysis. The quantitative assessment of these three health indicators is done using three metrics, i.e., monotonicity [5,7,44], robustness [7,44], and trendability [5,7,44,45]. Monotonicity measures whether the HI is monotonically increasing or decreasing. It is measured using Equation (4):

(4) $$Mon(X)=\frac{1}{K-1}\left|No.of\;d/dx>0-No.of\;d/dx<0\right|$$

Here, $X={\left\{x_{k}\right\}}_{k=1:K}$ is the sequence of values of the HI, $x_{k}$ is the value of the HI at time $t_{k}$, K is the total number of HI values in sequence X, and $d/dx=x_{k+1}-x_{k}$ is the gradient of the HI. The value of the monotonicity ranges from 0 to 1. For a bearing HI, higher values of monotonicity are desirable. Table 1 presents the values of monotonicity for the three health indicators, mentioned earlier, which are extracted from the IMS and PRONOSTIA datasets discussed in Section 2. It can be clearly observed in Table 1 that the proposed method for the construction of a bearing HI yields better monotonicity values for all bearings as compared to the other two health indicators.

The robustness metric measures how robust the HI is to random fluctuations, which may arise due to sensor noise, the stochasticity of bearing degradation, or variations in operating conditions. Robustness is measured using Equation (5):

(5) $$Rob(X)=\frac{1}{K}\sum_{k=1}^{K}\exp\left(-\left|\frac{x_{k}-x_{k}^{T}}{x_{k}}\right|\right)$$

Here, *K* is the total number of HI values, *x_k_* is the value of the HI at time *t_k_*, and $x_{k}^{T}$ is the mean trend value of the HI at time *t_k_*, which is usually calculated using a smoothing technique.

Table 2 presents the values of robustness for the three health indicators, mentioned earlier, which are extracted from the IMS and PRONOSTIA datasets discussed in Section 2. It can be clearly observed in Table 2 that the proposed method for the construction of a bearing HI yields better robustness values for all bearings as compared to the other two health indicators.

Similarly, the trendability metric measures the correlation between the HI and time. It is calculated using Equation (6):

(6) $$Tre(X,T)=\frac{K\left(\sum_{k=1}^{K}x_{k}t_{k}\right)-\left(\sum_{k=1}^{K}x_{k}\right)\left(\sum_{k=1}^{K}t_{k}\right)}{\sqrt{\left[K\sum_{k=1}^{K}x_{k}^{2}-{\left(\sum_{k=1}^{K}x_{k}\right)}^{2}\right]\left[K\sum_{k=1}^{K}t_{k}^{2}-{\left(\sum_{k=1}^{K}t_{k}\right)}^{2}\right]}}$$

Here, *t_k_* is the *k*-th value of time and *x_k_* is the value of the HI at time *t_k_*. The trendability value ranges from −1 to +1, where −1 indicates a strong negative correlation and +1 indicates a strong positive correlation. In this case, a strong positive correlation (or generally positive values of trendability) is desirable. Table 3 presents the values of trendability for the three health indicators, mentioned earlier, which are extracted from the IMS and PRONOSTIA datasets discussed in Section 2. It can be clearly observed in Table 3 that except for one bearing the proposed method for the construction of a bearing HI yields better trendability values for all bearings as compared to the other two health indicators.

Moreover, the HI curves obtained using the proposed method have also been used to estimate the RUL of bearings; these results have been compared to the ones obtained using conventional RMS. The purpose of constructing any health indicator is to use it to estimate the RUL of bearings. A good HI is easier to model and results in better RUL estimates. In order to model the behavior of the proposed HI and estimate the RUL of bearings, we used the adaptive regression models-based approach proposed in [2]. The prognostic performance achieved by the proposed HI is compared with the model-based approaches used by Li et al. [8] (i.e., the Paris model-based approach, exponential model-based approach, and improved exponential model-based approach), as well as the adaptive regression models-based approach proposed by Ahmad et al. [2]. Both Li et al. and Ahmad et al. used conventional RMS values as bearing HIs.

The prognostic performance of all these methods is measured using their cumulative relative accuracy (CRA) scores [14,15], which are given in Table 2, Table 3 and Table 4 for four bearings of the PRONOSTIA dataset. The CRA is the normalized and weighted sum of the relative accuracies of the RUL estimates at specific time instances [14,15]. The relative accuracy (RA) is a measure of error in the estimated value of RUL $r\left(i_{\lambda }\right)$ relative to the true value of the RUL $r_{*}\left(i_{\lambda }\right)$ at a specific time index $i_{\lambda }$ [13,14]. It is calculated using Equation (7):

(7) $$RA_{\lambda }=1-\frac{\left|r_{*}\left(i_{\lambda }\right)-\langle r\left(i_{\lambda }\right)\rangle \right|}{r_{*}\left(i_{\lambda }\right)}$$

Here, λ is the time window modifier, such that $t_{\lambda }=t_{p}+\lambda (t_{EOL}-t_{P})$, where $t_{p}$ refers to the time index when the RUL is estimated for the first time. Additionally, $t_{EOL}$ refers to the time index when the value of the estimated HI crosses the failure threshold. Table 2, Table 3 and Table 4 show that, with the exception of bearing 14, the proposed HI yields better CRA scores and better prognostic performance.

## 5. Conclusions

In this paper, an attempt was made to address an important problem in bearing health prognosis, i.e., the construction of a reliable HI to determine the progression of degradation in bearings. Bearing degradation is generally indirectly determined or inferred through different features of the bearing fault signals as it is prohibitively difficult to determine it directly through the direct assessment of a bearing’s condition. Once initiated, bearing degradation is assumed to be irreversible and a good bearing HI is expected to correlated well with this progressively increasing bearing degradation. Nevertheless, bearing fault signals are subject to noise and random fluctuations, which can affect the behavior and an HI and lead to poor inference about a bearing’s health condition. Hence, an HI is also expected to be robust to noise and random fluctuations. These characteristics also make it easier to model the trends in the HI over the lifetime of the bearing, which can lead to better RUL estimates. Hence, a new approach was presented for the construction of a bearing’s HI that considers the non-stationary nature of the vibration acceleration signals, which is often ignored during the construction of HIs for bearing health prognosis and may result in the loss of information that may be critical to quantifying a bearing’s degradation. Thus, instead of quantifying bearing degradation using a single value of the HI for the entire raw vibration acceleration signal, we proposed to quantify bearing damage by analyzing the raw signal at different resolutions. Therefore, the vibration acceleration signal is decomposed into different sub-bands, and the HI is extracted from individual sub-band signals, thereby enabling the evaluation of the original signal at different time-frequency resolutions. The HI curves extracted from each sub-band signal are smoothened using locally weighted regression and then evaluated using the proposed gradient-based metric at each time index. The HI curves with the maximum gradient are iteratively accumulated to render an HI with an optimal trend over the entire life of the bearing. The proposed method was tested on the IMS and PRONOSTIA datasets that are used as benchmark datasets for bearing health prognosis. The results indicated that unlike the traditional methods of calculating a bearing’s HI, the proposed method yields an HI that is more reflective of bearing degradation, i.e., it has better monotonicity and better correlates with bearing degradation. Moreover, it is more robust to noise and random fluctuations in measurement data. Furthermore, the resultant HI is easier to model and yields better prognostic performance when used to estimate the RUL of bearings.

## Figures and Tables

**Table 1:** Quantitative Evaluation of the Health Indicators using Monotonicity.

S. No.	Bearing	Health Indicators		
		Traditional RMS	Variance	Proposed
1	IMS Outer	0.0132	0.001	0.9868
2	IMS Inner	0.0144	0.0292	0.9133
3	IMS Roller	4.64 × 10^−4^	2.09 × 10^−2^	0.9995
4	PRONOSTIA b11	0.0071	0.0093	0.9925
5	PRONOSTIA b13	0.0152	0.4278	0.9836
6	PRONOSTIA b14	3.99 × 10^−2^	4.29 × 10^−1^	0.9629
7	PRONOSTIA b15	8.12 × 10^−4^	3.48 × 10^−1^	0.9736
8	PRONOSTIA b16	0.0037	0.3509	0.9163
9	PRONOSTIA b17	0.0053	0.378	0.9588
10	PRONOSTIA b22	0.0402	0.0402	0.9686
11	PRONOSTIA b23	0.0082	0.3335	0.9043
12	PRONOSTIA b24	0.0027	0.3675	0.9694
13	PRONOSTIA b25	0.0147	0.3323	0.9883
14	PRONOSTIA b26	0.0057	0.2767	0.9472
15	PRONOSTIA b27	0.0565	0.2826	0.9609
16	PRONOSTIA b32	0.0073	0.0024	0.9383
17	PRONOSTIA b33	0.0438	0.4539	0.9977

**Table 2:** Quantitative Evaluation of the Health Indicators using Robustness.

S. No.	Bearing	Health Indicators		
		Traditional RMS	Variance	Proposed
1	IMS Outer	0.9487	0.944	0.9958
2	IMS Inner	0.9685	0.9586	0.9954
3	IMS Roller	0.9754	0.9737	0.9947
4	PRONOSTIA b11	0.9467	0.8936	0.9968
5	PRONOSTIA b13	0.9362	0.8702	0.9968
6	PRONOSTIA b14	0.962	0.8181	0.9974
7	PRONOSTIA b15	0.9675	0.9448	0.9982
8	PRONOSTIA b16	0.9347	0.8825	0.9981
9	PRONOSTIA b17	0.9635	0.8563	0.9983
10	PRONOSTIA b22	0.9699	0.9395	0.9979
11	PRONOSTIA b23	0.9073	0.8735	0.9924
12	PRONOSTIA b24	0.9478	0.7794	0.9971
13	PRONOSTIA b25	0.9446	0.9172	0.9985
14	PRONOSTIA b26	0.9611	0.9431	0.9981
15	PRONOSTIA b27	0.9402	0.9112	0.9947
16	PRONOSTIA b32	0.9352	0.8618	0.9974
17	PRONOSTIA b33	0.9622	0.8908	0.9983

**Table 3:** Quantitative Evaluation of the Health Indicators using Trendability.

S.No.	Bearing	Health Indicators		
		Traditional RMS	Variance	Proposed
1	IMS Outer	0.668	0.3965	0.8131
2	IMS Inner	0.3882	0.2689	0.7833
3	IMS Roller	0.8465	0.7931	0.9572
4	PRONOSTIA b11	0.6798	0.3792	0.7819
5	PRONOSTIA b13	0.55	0.8353	0.6793
6	PRONOSTIA b14	0.7095	0.7156	0.7158
7	PRONOSTIA b15	−0.1582	−0.4051	0.6654
8	PRONOSTIA b16	−0.0922	−0.2725	0.9
9	PRONOSTIA b17	0.336	0.4472	0.8316
10	PRONOSTIA b22	0.745	0.5847	0.9623
11	PRONOSTIA b23	0.0211	−0.4844	0.4235
12	PRONOSTIA b24	−0.0938	−0.3742	0.8288
13	PRONOSTIA b25	−0.6617	−0.7458	0.9304
14	PRONOSTIA b26	−0.0114	−0.2822	0.6057
15	PRONOSTIA b27	0.248	0.2095	0.4489
16	PRONOSTIA b32	0.1087	0.1468	0.8077
17	PRONOSTIA b33	0.6403	0.6584	0.8543

**Table 4:** The CRA Scores obtained using the Proposed HI and other RUL estimation methods.

Method	Bearing 11	Bearing 13	Bearing 14	Bearing 15
Paris Model	0.6967	0.6074	0.6317	0.7443
Exponential Model	0.7111	0.5311	0.5420	0.7463
Improved Exponential Model	0.8696	0.7623	0.8712	0.9324
Adaptive Predictive Model	0.9362	0.9003	0.9608	0.7790
Proposed	0.9395	0.9287	0.7623	0.9547
